# Association Between Average Daily Television Viewing Time and Chronic Obstructive Pulmonary Disease-Related Mortality: Findings From the Japan Collaborative Cohort Study

**DOI:** 10.2188/jea.JE20140185

**Published:** 2015-06-05

**Authors:** Shigekazu Ukawa, Akiko Tamakoshi, Hiroshi Yatsuya, Kazumasa Yamagishi, Masahiko Ando, Hiroyasu Iso

**Affiliations:** 1 Department of Public Health, Hokkaido University Graduate School of Medicine, Sapporo, Japan; 1 北海道大学大学院医学研究科社会医学講座公衆衛生学分野; 2 Department of Public Health, Fujita Health University, Toyoake, Aichi, Japan; 2 藤田保健衛生大学医学部公衆衛生学講座; 3 Department of Public Health Medicine, Faculty of Medicine, University of Tsukuba, Tsukuba, Ibaraki, Japan; 3 筑波大学医学医療系社会健康医学研究室; 4 Center for Advanced Medicine and Clinical Research, Hospital, Nagoya University, Nagoya, Japan; 4 名古屋大学医学部附属病院先端医療・臨床研究支援センター; 5 Public Health, Department of Social and Environmental Medicine, Osaka University Graduate School of Medicine, Suita, Osaka, Japan; 5 大阪大学大学院医学系研究科社会環境医学講座

**Keywords:** chronic obstructive pulmonary disease, sedentary behavior, obstructive lung disease, cohort study, risk assessment

## Abstract

**Background:**

Sedentary behavior is associated with cardiovascular disease, diabetes mellitus, and cancer morbidity, and watching television (TV) is an important sedentary behavior. The aim of this study is to clarify the association between TV viewing time and chronic obstructive pulmonary disease (COPD)-related mortality in Japanese adults.

**Methods:**

Using the Cox proportional hazard model, we assessed COPD-related mortality by TV viewing time in a national cohort of 33 414 men and 43 274 women without cancer, stroke, myocardial infarction, or tuberculosis at baseline (1988–1990).

**Results:**

The median follow-up was 19.4 years; 244 men and 34 women died of COPD. Men watching ≥4 hours/day of TV were more likely to die of COPD than those watching <2 hours/day (hazard ratio 1.63; 95% confidence interval, 1.04–2.55), independent of major confounders. No association was found in women.

**Conclusions:**

Avoiding a sedentary lifestyle, particularly prolonged TV viewing, may help in preventing death from COPD among men.

## Abstract (ja)

**【背景】:**

テレビ（TV）視聴は、座位活動の代表的なものである。長い座位時間は、心血管疾患や糖尿病、およびいくつかのがんのリスク要因とされる。本研究の目的は、TV視聴時間と慢性閉塞性肺疾患（COPD）死亡との関連を明らかにすることである。

**【方法】:**

がん、脳卒中、心筋梗塞、結核の既往歴のない40から79歳の76,688(男性33,414、女性43,274)人を本研究の対象者とした。テレビ視聴時間とCOPD死亡との関連を、男女別にCox比例ハザードモデルによりハザード比と95%信頼区間（95%CI）を算出した。

**【結果】:**

追跡期間（中央値：19.4年）のうち278（男性244、女性34）名がCOPDにより死亡した。男性では、2時間未満のテレビ視聴者と比較して4時間以上では1.63（95%CI: 1.04-2.55）倍と1日4時間以上のテレビ視聴者においてCOPD死亡のハザード比が有意に高かった。女性ではテレビ視聴時間とCOPD死亡との間には有意な関連はなかった。

**【結論】:**

男性において、TV視聴のような座位行動を避ける事で、COPDによる死亡を予防できる可能性が示唆された。

## INTRODUCTION

Chronic obstructive pulmonary disease (COPD) is characterized by a chronic abnormal inflammatory response in the lung and an accelerated decline in lung function.^1^ In Japan, the prevalence of COPD is reportedly 10.9% among people aged >40 years,^2^ with worldwide estimates of 8.5%–22.2%.^3^ Cigarette smoking,^4^ air pollution (such as smoke from biomass fuel),^5^ occupational exposure to toxic gas or dust,^6^ and pulmonary tuberculosis infection^7^ are all documented risk factors for COPD.

Sedentary behavior, the absence of moderate-vigorous physical activity,^8^ is known to cause poor health and is associated with cardiovascular disease, diabetes mellitus, and cancer.^9^^,^^10^ Watching TV is a particularly important sedentary behavior^11^ that has been related to an increased risk of cardiovascular disease^12^ and several cancers, including lung cancer.^13^ However, whether it is associated with COPD-related mortality remains unclear. Therefore, we examined the association between the average TV viewing duration per day (daily TV viewing time) and COPD-related mortality in Japanese adults in a large-scale national cohort study.

## MATERIAL AND METHODS

### Study population

The Japan Collaborative Cohort Study for Evaluation of Cancer Risk (JACC Study) was established in 1988–90 and has been described in detail elsewhere.^14^ Briefly, we enrolled 110 585 apparently healthy inhabitants (46 395 men and 64 190 women) aged 40–79 years from 45 areas throughout Japan.

### Data collection

Participants were recruited at the time of their health check-up and assessed using a self-administered questionnaire, and the response rate was 86%–91%. Information on the average daily TV viewing time in hours was obtained via the question, “How many hours a day, on an average, do you spend watching TV?” The average daily TV viewing time was categorized into the following three groups: <2 hours/day, 2 to <4 hours/day, and ≥4 hours/day. We also collected information on age, study area, smoking status, environmental tobacco smoke (ETS) exposure, body mass index (BMI; <18.5, 18.5–24.9, or ≥25.0 kg/m^2^, or unknown), education level (junior high school, high school, college diploma, or unknown), marital status (single, married, divorced/widowed, or unknown), alcohol consumption (never, former, current alcohol drinker, or unknown), and time spent exercising (≥5, 3–4, or 1–2 hours/week, almost none, or unknown).

Smoking status was first classified as never, former, and current smoking. Never smokers were further classified by the level of ETS exposure at home or in public places, including public halls, the workplace, or public transportation (almost every day, <4 days a week, or unknown). Former smokers were further classified by the time since they quit (≥10 or <10 years, or unknown). Current smokers were further classified by the number of pack years (≥20 or <20 pack years, or unknown). BMI was calculated with self-reported height and weight.

### Follow-up

The dates and causes of death of participants were confirmed by death certificates and coded according to the Tenth Revision of the International Classification of Disease (ICD-10). The primary outcome in the present study was the underlying cause of death in patients with COPD (J41–44 or J47). The overall study design was approved by the Ethics Review Committee of Nagoya University School of Medicine.

### Statistical analysis

Sex-specific multivariate hazard ratios (HRs) and confidence intervals (CIs) for COPD-related mortality were calculated using a Cox proportional hazards model. Age (as a continuous variable), study area, smoking status, ETS exposure, BMI, educational level, marital status, alcohol consumption, and exercise time were included in the multivariate models. We also conducted additional analysis to assess the associations between the average daily TV viewing time in hours and the risk of COPD-related mortality. Analyses were repeated by restricting to current smokers and by excluding participants who died or were censored within 5 years from the study baseline. An alpha level of 0.05 was considered statistically significant. All statistical analyses were performed using SAS 9.4 (SAS Institute Inc., Cary, NC, USA).

## RESULTS

Of the 110 585 original cohort members, 8909 participants were excluded because they had a medical history of cancer, stroke, myocardial infarction, or tuberculosis at baseline. An additional 14 669 participants from 5 areas were excluded because their questionnaires did not include the specified question. In addition, we excluded a further 7521 and 2798 participants with data missing for smoking status and average daily TV viewing times, respectively. Accordingly, 76 688 participants (33 414 men and 43 274 women) were analyzed in the present study. Participants who moved out from the study area during the study period were treated as censored cases (*n* = 2976).

The mean (standard deviation [SD]) age of participants at baseline was 56.9 (10.1) years (males: 56.7 [10.1] years; females: 57.0 [10.1] years). Table 1 summarizes the baseline characteristics of participants according to the average daily TV viewing time. Compared with men and women who viewed TV for <2 hours/day, those who viewed TV for ≥4 hours/day tended to be older, were more likely to be a smoker and less likely to have a normal BMI or college diploma. Men who viewed TV for >4 hours/day were more likely to be married and less likely to be current drinkers than men who viewed TV for <2 hours/day. The opposite was true for women.

**Table 1.** Baseline characteristics of participants by television viewing duration

Characteristic	Category	Males			Females		
	
		Television viewing time (hours/day)			Television viewing time (hours/day)		
	
		<2 (*n* = 6632)	≥2 to <4 (*n* = 20 013)	≥4 (*n* = 6769)	<2 (*n* = 8804)	≥2 to <4 (*n* = 23 267)	≥4 (*n* = 11 203)
Age (SD), years		55.5 (10.3)	56.1 (9.9)	60.0 (10.0)	55.0 (10.3)	56.4 (9.8)	59.8 (9.7)
Smoking status							
Never smokers’ exposure to ETS at home or in public places	Less than 4 days a week	444 (6.7)	1082 (5.4)	219 (3.2)	2867 (32.5)	7854 (33.8)	3092 (27.6)
	Almost everyday	297 (4.5)	871 (4.4)	297 (4.4)	1360 (15.5)	4458 (19.1)	2343 (20.9)
	Unknown	1000 (15.0)	2157 (10.8)	602 (8.9)	4200 (47.6)	9616 (41.3)	4679 (41.8)
Former smoker	Quit ≥10 years ago	754 (11.4)	2590 (12.9)	918 (13.6)	47 (0.5)	157 (0.7)	138 (1.2)
	Quit <10 years ago	725 (11.0)	2206 (11.0)	803 (11.9)	35 (0.4)	102 (0.4)	77 (0.7)
	Unknown	43 (0.7)	137 (0.7)	45 (0.7)	7 (0.1)	18 (0.1)	19 (0.2)
Current smoker	<20 pack years	6 (0.1)	12 (0.1)	4 (0.1)	3 (0.1)	8 (0.1)	9 (0.1)
	≥20 pack years	3314 (49.9)	10 829 (54.1)	3827 (56.4)	269 (3.1)	1008 (4.3)	819 (7.3)
	Unknown	49 (0.7)	129 (0.6)	54 (0.8)	16 (0.2)	46 (0.2)	27 (0.2)
Body mass index (kg/m^2^)	<18.5	334 (5.3)	868 (4.5)	410 (6.4)	594 (7.1)	1283 (5.8)	609 (5.8)
	18.5–24.9	4816 (76.8)	14 431 (75.2)	4701 (73.3)	6137 (73.3)	15 813 (70.9)	7025 (66.4)
	≥25.0	1123 (17.9)	3892 (20.3)	1303 (20.3)	1637 (19.6)	5204 (23.3)	2939 (27.8)
College education	Yes	3849 (58.0)	11 881 (59.4)	3706 (54.8)	5681 (64.5)	14 685 (63.1)	6441 (57.5)
Married	Yes	5213 (78.6)	17 240 (86.1)	5524 (81.6)	6502 (73.9)	18 477 (79.4)	7936 (70.8)
Current alcohol drinker	Yes	5085 (76.7)	14 972 (74.8)	4414 (65.2)	1810 (20.6)	4933 (21.2)	2458 (21.9)
Weekly exercise time (hours/week)	≥5	357 (6.3)	1348 (7.1)	482 (7.5)	338 (4.5)	950 (4.3)	490 (4.7)
	3–4	358 (6.4)	1395 (7.3)	452 (7.1)	352 (4.7)	1188 (5.4)	619 (5.9)
	1–2	987 (17.5)	3290 (17.2)	988 (15.4)	969 (12.8)	3075 (14.0)	1454 (13.9)
	Almost none	3937 (69.8)	13 074 (68.4)	4484 (70.0)	5902 (78.0)	16 803 (76.3)	7881 (75.5)

Values are expressed as mean ± standard deviation or number (%).

ETS, environmental tobacco smoke; SD, standard deviation.

During the median follow-up period of 19.4 years, 278 participants (244 men and 34 women) died of COPD. Of the original population, 4415 participants left the study area and 17 283 participants died from causes other than COPD. The HRs for COPD-related mortality associated with the average daily TV viewing time are shown in Table 2. Compared with men who watched TV for less than 2 hours/day, those who watched TV for more than 4 hours/day were more likely to die of COPD (HR 1.63; 95% CI, 1.04–2.55). This finding was independent of age, study area, smoking status, BMI, education, marital status, alcohol consumption, and exercise time. The average daily TV viewing time was linearly and positively associated with COPD-related mortality (*P* = 0.04). Similarly, elevated HRs were observed in a sensitivity analysis that excluded participants who died or were censored within 5 years of the study baseline (HR 1.68; 95% CI, 1.03–2.74) and in an analysis restricted to men who were current smokers (HR 1.70; 95% CI, 0.98–2.97). However, the average daily TV viewing time was not associated with COPD-related mortality in women.

**Table 2.** Hazard ratios for chronic obstructive pulmonary disease-related mortality associated with television viewing duration

Television viewing time (hours/day)	Person-years	Cases	Age and study area adjusted HR (95% CI)	Multivariate HR (95% CI)^a^	Multivariate HR (95% CI)^b^
Men					
<2	109 892	35	ref	ref	ref
≥2 to <4	327 148	136	1.51 (1.03–2.20)*	1.37 (0.93–2.00)	1.40 (0.92–2.14)
≥4	100 167	73	1.87 (1.23–2.82)*	1.63 (1.08–2.47)*	1.63 (1.04–2.55)*
*P* for trend			0.005	0.02	0.04
Women					
<2	153 031	6	ref	ref	ref
≥2 to <4	398 006	18	1.18 (0.46–3.10)	1.08 (0.41–2.80)	1.03 (0.40–2.75)
≥4	178 265	10	1.00 (0.35–2.88)	0.85 (0.30–2.45)	0.84 (0.29–2.48)
*P* for trend			0.43	0.89	0.35
**For current smokers**					
Men					
<2	55 528	23	ref		ref
≥2 to <4	178 255	91	1.35 (0.85–2.16)		1.37 (0.82–2.30)
≥4	57 983	50	1.68 (1.01–2.75)*		1.70 (0.98–2.97)
*P* for trend			0.09		0.13
Women					
<2	4871	1	ref		ref
≥2 to <4	17 467	3	0.71 (0.06–7.51)		0.44 (0.03–6.63)
≥4	12 728	3	0.91 (0.08–9.83)		1.14 (0.09–15.34)
*P* for trend			0.18		0.93
**Excluding participants who died or were censored within 5 years from baseline**					
Men					
<2	108 921	31	ref	ref	ref
≥2 to <4	324 101	128	1.62 (1.08–2.42)	1.46 (0.97–2.18)	1.53 (0.97–2.40)
≥4	98 552	64	1.89 (1.22–2.94)*	1.67 (1.06–2.56)*	1.68 (1.03–2.74)*
*P* for trend			0.01	0.06	0.09
Women					
<2	152 244	6	ref	ref	ref
≥2 to <4	396 192	16	0.98 (0.37–2.61)	0.90 (0.34–2.38)	0.91 (0.33–2.47)
≥4	176 663	9	0.85 (0.29–2.50)	0.72 (0.24–2.11)	0.75 (0.25–2.28)
*P* for trend			0.38	0.23	0.33

HR, hazard ratio; CI, confidence interval.

*P < 0.05.

^a^Adjusted for age, study area, and smoking status (never smokers: exposure to environmental tobacco smoke at home or in public places almost every day or less or unknown, former smokers: time since quitting ≥10 or <10 years or unknown, and current smokers: ≥20 or <20 pack years or unknown).

^b^Further adjusted for body mass index (<18.5, 18.5–24.9, or ≥25.0 kg/m^2^ or unknown), education (junior high school, high school, college diploma, or unknown), marital status (single, married, divorced/widowed, or unknown), alcohol drinking (never, former, current alcohol drinker, or unknown), exercise time (≥5, 3–4, 1–2 hours/week, almost none, or unknown). Smoking status was not adjusted for in current smokers. *P* for trend: associations between the original continuous variables of daily hours spent watching television and risk of chronic obstructive pulmonary disease-related mortality.

## DISCUSSION

In this large cohort study, we found that a longer average daily TV viewing time was associated with an increased risk of mortality among Japanese men with COPD after adjusting for potential confounders. This association persisted even after excluding participants who died from other causes or were censored. However, similar associations did not exist among women.

To our knowledge, this is the first report to investigate the association between the average daily TV viewing time and COPD-related mortality. The associations were similar to those in a previous study that reported prolonged daily TV viewing time as a risk factor for lung cancer.^13^ COPD is characterized by a chronic abnormal inflammatory response in the lung.^1^ Although potential mechanisms underlying the increase in COPD-related mortality with an increase in the average daily TV viewing time remain unclear, we assume that it is the sedentary nature of watching TV. Consistent with this theory, a longer average daily TV viewing time is reported to be a marker of sedentary behavior,^15^ and prolonged sedentary behavior has been shown to increase the inflammatory factors interleukin-6, tumor necrosis factor-alpha, and leptin,^16^^,^^17^ all of which are known to be risk factors for susceptibility to lung function impairment.^18^^–^^20^ Sedentary behavior also causes metabolic dysfunction,^21^ which can lead to hyperinsulinemia that can modify cell differentiation, apoptosis, and proliferation.^22^ These factors may contribute to lung dysfunction; however, further biological or epidemiological studies are needed to clarify this issue.

In this study, estimated HRs were comparable among analyses of all male participants and those restricted to current male smokers. However, none of the associations reached statistical significance. We consider this result to be due to the lack of statistical power.

We found an association between COPD-related mortality and average daily TV viewing time for male participants only. This may be related to the fact that men reportedly only spend 1 hour/day on housework, whereas women reportedly spend 4 to 5 hours/day on housework, with more than 1.3 hours/day spent on housework completed while watching TV.^23^ Therefore, sedentary time may be overestimated, and the average daily TV viewing time may not be an appropriate indicator of sedentary behavior for women in Japan.

The major strengths of this study are its prospective cohort design, the inclusion of participants from throughout Japan, and its long follow-up period. However, several limitations should be noted. First, we obtained information on the average daily TV viewing time through self-reporting that was not validated; therefore, some misclassification may have been included in our results. However, this would have occurred randomly because participants could not foresee subsequent events at baseline. Accordingly, such an error may have diminished the estimated HR. Second, some participants with COPD at baseline might be included in this cohort; therefore, a potential reverse relationship between COPD and sedentary behavior could have been present. However, exclusion of participants who died or were censored within 5 years of the study baseline did not materially affect the results. In addition, information on potential confounders for COPD was collected at baseline and adjusted as much as possible in the analysis. Third, sedentary behavior is characterized by an energy expenditure less than 1.5 multiples of the basal metabolic rate, including activities such as computer use, occupational sitting, reading, and driving a car^24^; therefore, TV viewing time is not always a good indicator of total sedentary time. Using an objective measurement, such as an accelerometer, would make our results more robust.^25^

In conclusion, this cohort study indicated that an average daily TV viewing time of >4 hours/day was associated with an increased risk of COPD-related mortality among men aged 40–79 years in Japan compared with the risk associated with a viewing time of <2 hours/day. Our findings suggest that avoiding a sedentary lifestyle by decreasing the average daily TV viewing time may help in preventing death from COPD among men.

## ONLINE ONLY MATERIAL

Abstract in Japanese.

## APPENDIX

### Members of the Japan Collaborative Cohort Study Group

The present members of the JACC Study Group who coauthored this paper include Dr. Akiko Tamakoshi (present chairperson of the study group), Hokkaido University Graduate School of Medicine; Drs. Mitsuru Mori and Fumio Sakauchi, Sapporo Medical University School of Medicine; Dr. Yutaka Motohashi, Akita University School of Medicine; Dr. Ichiro Tsuji, Tohoku University Graduate School of Medicine; Dr. Yosikazu Nakamura, Jichi Medical School; Dr. Hiroyasu Iso, Osaka University School of Medicine; Dr. Haruo Mikami, Chiba Cancer Center; Dr. Michiko Kurosawa, Juntendo University School of Medicine; Dr. Yoshiharu Hoshiyama, Yokohama Soei University; Dr. Naohito Tanabe, University of Niigata Prefecture; Dr. Koji Tamakoshi, Nagoya University Graduate School of Health Science; Dr. Kenji Wakai, Nagoya University Graduate School of Medicine; Dr. Shinkan Tokudome, National Institute of Health and Nutrition; Dr. Koji Suzuki, Fujita Health University School of Health Sciences; Dr. Shuji Hashimoto, Fujita Health University School of Medicine; Dr. Shogo Kikuchi, Aichi Medical University School of Medicine; Dr. Yasuhiko Wada, Faculty of Nutrition, University of Kochi; Dr. Takashi Kawamura, Kyoto University Center for Student Health; Dr. Yoshiyuki Watanabe, Kyoto Prefectural University of Medicine Graduate School of Medical Science; Dr. Kotaro Ozasa, Radiation Effects Research Foundation; Dr. Tsuneharu Miki, Kyoto Prefectural University of Medicine Graduate School of Medical Science; Dr. Chigusa Date, School of Human Science and Environment, University of Hyogo; Dr. Kiyomi Sakata, Iwate Medical University; Dr. Yoichi Kurozawa, Tottori University Faculty of Medicine; Drs. Takesumi Yoshimura and Yoshihisa Fujino, University of Occupational and Environmental Health; Dr. Akira Shibata, Kurume University; Dr. Naoyuki Okamoto, Kanagawa Cancer Center; and Dr. Hideo Shio, Moriyama Municipal Hospital.
