# Supplementary material for: Association Between Average Daily Television Viewing Time and Chronic Obstructive Pulmonary Disease-Related Mortality: Findings From the Japan Collaborative Cohort Study
Source: J Epidemiol. 2015 Jun 5;25(6):431–6. doi: 10.2188/jea.JE20140185 (PMC4444497; doi:10.2188/jea.JE20140185)
Supplement: Abstract in Japanese. [file je-25-431-s001.pdf]

テレビ視聴時間と慢性閉塞性肺疾患（COPD）死亡との関連：JACC Study

鵜川重和[1]、玉腰暁子[1]、八谷寛[2]、山岸良匡[3]、安藤昌彦[4]、磯博康[5]

[1] 北海道大学大学院医学研究科社会医学講座公衆衛生学分野、[2] 藤田保健衛生大学医学部公衆衛生学講座、[3] 筑波大学医学医療系社会健康医学研究室、[4] 名古屋大学医学部附属病院先端医療・臨床研究支援センター、[5] 大阪大学大学院医学系研究科社会環境医学講座

【背景】テレビ（TV）視聴は、座位活動の代表的なものである。長い座位時間は、心血管疾患や糖尿病、およびいくつかのがんのリスク要因とされる。本研究の目的は、TV 視聴時間と慢性閉塞性肺疾患（COPD）死亡との関連を明らかにすることである。

【方法】 がん、脳卒中、心筋梗塞、結核の既往歴のない 40 から 79 歳の 76,688(男性 33,414、女性 43,274)人を本研究の対象者とした。テレビ視聴時間と COPD 死亡との関連を、男女別に Cox 比例ハザードモデルによりハザード比と 95%信頼区間（95%CI）を算出した。

【結果】追跡期間（中央値：19.4 年）のうち 278（男性 244、女性 34）名が COPD により死亡した。男性では、2 時間未満のテレビ視聴者と比較して 4 時間以上では 1.63（95%CI: 1.04-2.55）倍と 1 日 4 時間以上のテレビ視聴者において COPD 死亡のハザード比が有意に高かった。女性ではテレビ視聴時間と COPD 死亡との間には有意な関連はなかった。

【結論】男性において、TV 視聴のような座位行動を避ける事で、COPD による死亡を予防できる可能性が示唆された。
